# Improving Patient Safety Culture Perceptions in Resource‐Limited Operating Rooms: A Nurse‐Led Multifaceted Implementation of the WHO Surgical Safety Checklist

**DOI:** 10.1155/jonm/4718057

**Published:** 2026-09-30

**Authors:** Wiem Aouicha, Mohamed Ayoub Tlili, Bushra Alshammari, Hamdi Lamine, Nahed Mousa Saber, Nojoud Abdullah Alrashidi, Amany Ali Abd Elsalam, Hadya Obied Alenzi, Samah Ramadan Ibrahim Elrefaey, Mohammad Alboliteeh, Houyem Said Latiri, Manel Mallouli, Areej Ali Fahad Alshammary, Hind mobark fahd Alshamry, Maha Sanat Alrashedi, Nick Sevdalis, Aziza Zakaria Faramawy Ali

**Affiliations:** ^1^ Nursing Administration Department College of Nursing University of Hail Hail Saudi Arabia uoh.edu.sa; ^2^ Medical Surgical Nursing Department College of Nursing University of Hail Hail 2440 Saudi Arabia uoh.edu.sa; ^3^ Community Health Nursing Department College of Nursing University of Hail Hail Saudi Arabia uoh.edu.sa; ^4^ Maternal and Child Health Nursing Department College of Nursing University of Hail Hail Saudi Arabia uoh.edu.sa; ^5^ Psychiatric and Mental Health Nursing Department College of Nursing Benha University Benha Egypt bu.edu.eg; ^6^ Community and Mental Health Nursing Department College of Nursing Najran University Najran Saudi Arabia nu.edu.sa; ^7^ Faculty of Medicine of Sousse University of Sousse Sousse Tunisia uc.rnu.tn; ^8^ Laboratory of Research “LR12ES03” Faculty of Medicine of Sousse University of Sousse Sousse Tunisia uc.rnu.tn; ^9^ Aja Long Term Care Hospital & Rehabilitation Centre Ministry of Health Hail Saudi Arabia moh.gov.sa; ^10^ Centre for Behavioral and Implementation Science Interventions Yong Loo Lin School of Medicine National University of Singapore Singapore nus.edu.sg; ^11^ Nursing Administration Department Faculty of Nursing Benha University Benha Egypt bu.edu.eg

**Keywords:** implementation strategies, nurse champions, operating room, patient safety culture, resource-limited settings, surgical safety checklist

## Abstract

**Background:**

Strengthening patient safety culture (PSC) is essential for enhancing clinical practice, teamwork, and nursing leadership in surgical settings. However, evidence on interventions that promote PSC in operating rooms (ORs) in low‐ and middle‐income countries remains limited.

**Objective:**

To evaluate the association between a multifaceted intervention and PSC perceptions among OR professionals in a resource‐limited setting.

**Methods:**

A quasiexperimental, nonrandomized before‐and‐after study with a concurrent control group was conducted over 21 months across 12 ORs in two tertiary hospitals in Tunisia. One hospital served as the intervention site and the other as the control site. All eligible healthcare professionals (*n* = 347; surgeons, anesthesiologists, nurses, OR specialized nurses, and assistant caregivers) were invited to participate. The intervention focused on implementing the Surgical Safety Checklist (SSC) and included team‐based training, practice runs, awareness campaigns, local nurse champions, continuous mentorship, and audit and feedback processes. Data were collected at three time points (baseline, postintervention, and follow‐up) using the French version of the Hospital Survey on PSC. All data were anonymized to ensure confidentiality.

**Results:**

The intervention was associated with significant and sustained improvements in four key dimensions of PSC: “Organizational learning–continuous improvement” increased from 37.7% to 50.6%, “Teamwork within units” from 47.6% to 59.1%, “Communication openness” from 27.2% to 46.2%, and “Teamwork across units” from 29.2% to 37.5%. The overall perceived level of safety increased from 70.7% at baseline to 82.4% postintervention and was sustained at follow‐up.

**Conclusions:**

A multifaceted intervention focusing on SSC implementation and incorporating team‐based training, continuous feedback, and supported practice was associated with improved PSC perceptions in a resource‐limited setting. Such approaches may support the sustainable integration of safety protocols into routine surgical practice.

**Implications for Nursing Management:**

Findings highlight the critical role of nurse champions and the active engagement of OR managers in leading safety initiatives. Structured implementation support, including mentorship, continuous training, and audit and feedback, may facilitate adherence to the SSC. For perioperative nursing leaders in resource‐limited settings, strong leadership engagement and organizational support are essential to sustain long‐term implementation of safe surgical practices.

## 1. Introduction

The operating room (OR) is a complex and dynamic environment characterized by a very complex system that requires a highly skilled multidisciplinary team to provide optimal surgical care. ORs present multiple factors that increase the risk of errors [1, 2]. Several studies have demonstrated that a large number of adverse events (AEs) occur in the OR [3–5]. An earlier systematic review by De Vries et al., including over 70,000 inpatient medical records, revealed that the majority of AEs (39.6%) were linked to surgical procedures [6]. With regard to surgical safety, the situation in low‐ and middle‐income countries (LMICs) is much worse [7]. Despite contributing a smaller share of all surgical operations performed worldwide, emerging nations contribute more to the global burden of poor surgical outcomes [7]. Surgical mortality in developing countries is ten times higher than in developed nations [8]. In Tunisia, Letaief et al. reported that 55% of AEs were associated with surgical care, 60% of which were deemed preventable [9].

According to the High Authority of Health, the origin of these AEs is rarely related to a lack of technical skills but rather to a lack of Patient Safety Culture (PSC) among caregivers [10]. This concept has been associated with improved clinical outcomes [11]. Numerous studies have explored the impact of PSC, particularly in ORs and surgical settings [12–16]. For instance, in the United States, a positive PSC was significantly associated with fewer surgical site infections in seven Minnesota community hospitals [12]. Another study conducted across 31 hospitals in South Carolina, involving 1793 OR professionals, reported that strong PSC was linked to a significant decrease in postoperative mortality [16]. From an economic perspective, a positive PSC was associated with reductions in mortality (44,000 fewer deaths) and economic losses (up to US$2.9 billion) [17, 18]. Two studies conducted in ORs in LMICs noted that PSC was underdeveloped in these settings and presented significantly lower scores in comparison to other research [19, 20]. One contributing factor may be the nonuse of the standardized Surgical Safety Checklist (SSC) in Tunisian surgical practice [21].

The Safe Surgery Saves Lives campaign, one of the most successful programs to reduce surgical complications, led to the development and widespread adoption of the World Health Organization (WHO) SSC that has become a prominent safety intervention globally over the past decade [22–24]. The first WHO trial of the SSC was conducted in eight hospitals globally and resulted in a substantial decrease in both complication and mortality rates [24]. Numerous systematic reviews have reported that SSC implementation has positive effects on patient outcomes, including reduced complications [25, 26], such as wound infections [26], blood loss [26], and mortality rates [25, 26]. In addition, research has shown that SSC improves communication and teamwork, reduces operating time, and may also reduce operating costs [27].

However, the implementation of the SSC has been found to be a challenge in some LMICs because of factors such as a lack of resources, a limited understanding of the importance of the checklist, and resistance to change [28, 29]. The WHO unsuccessfully attempted nationwide implementation of the SSC in 15 African countries in 2011 [30]. After one year, only one hospital in the 15 countries had managed to implement the SSC, and none had started a nationwide implementation [30].

In these resource‐limited settings, empowering nurse leaders is a critical, yet frequently overlooked, approach for supporting quality improvement initiatives. Because nurses provide continuous patient care in the OR, they are in a unique position to champion standardized protocols like the SSC and promote effective communication and teamwork for a robust safety culture [31].

Despite the long‐recognized positive association between PSC and patient outcomes [11], assessing and particularly improving PSC in the OR has been relatively neglected and underexplored [32]. Furthermore, although checklist implementation has been proposed as a strategy to enhance patient safety, implementation efforts in resource‐constrained settings remain limited and sporadic [33].

The Donabedian’s structure–process–outcome (SPO) model and knowledge to action framework (KTA) provided the theoretical framework for this study. According to the SPO model, the organizational structure and care delivery processes affect health outcomes [34, 35]. SSC implementation represents a structural intervention that seeks to institutionalize a commitment to continuous improvements in patient safety outcomes (outcome), surgical processes (process), and organizational structures (structure). The KTA framework emphasizes translating knowledge into practice through education, local context adaptation, barrier identification, and outcome evaluation [36]. Taken together, these two frameworks support the use of continuous education and shared training to implement the SSC as a multifaceted system‐level intervention to improve PSC and guide the design, implementation, and evaluation of the study.

Owing to the limited attempts to implement the SSC in LMICs and the relative lack of attention given to improving PSC, particularly in ORs, our study aimed to evaluate the association between a multifaceted intervention and PSC perceptions among OR professionals in a resource‐limited setting (at a tertiary care hospital in Tunisia).

Specifically, the study sought to (1) assess changes in PSC dimensions following the multifaceted intervention; (2) examine changes in overall perceived patient safety levels among OR staff; (3) evaluate the sustainability of changes over time; and (4) compare PSC outcomes between intervention and control hospitals.

## 2. Methods

### 2.1. Study Design and Setting

This study used a quasiexperimental, nonrandomized before‐and‐after design with a concurrent control group and was conducted over 21 months. It included two independent groups: an intervention group and a control group, each corresponding to a different hospital. Data were collected at three time points. The baseline assessment (M1) was conducted during the first two months, after which the intervention was implemented over the following 15 months, until April 2021. The postintervention assessment (M2) was conducted in May 2021, followed by a follow‐up assessment (M3) four months later to track changes over time.

The study was conducted in two public tertiary hospitals in the region of Sousse, Tunisia, specifically in the ORs that agreed to participate. The two hospitals were comparable tertiary care institutions in terms of capacity, range of surgical specialties, and organizational structure. Allocation of hospitals to the intervention and control groups was determined by practical and organizational considerations, including administrative approval, accessibility, and readiness to implement the intervention, rather than through randomization. Sahloul University Hospital, which served as the intervention site, is a healthcare facility with a capacity of 688 beds and eight surgical units. In contrast, Farhat Hached University Hospital represented the control group, with a capacity of 600 beds and four surgical units covering different surgical specialties.

To enhance comparability and minimize potential sources of bias, both groups underwent identical data collection procedures with the same measurement tools and assessment timelines (M1, M2, and M3).

### 2.2. Study Population

The target population consisted of all eligible OR professionals (physicians, nurses, OR specialized nurses, and assistant caregivers) of Sahloul and Farhat Hached University Hospitals.

An exhaustive recruitment approach was adopted and targeted all healthcare professionals (*n* = 347) who were employed in the 12 ORs in the aforementioned hospitals, without sampling. The inclusion and noninclusion criteria were set based on the recommendations listed in the User’s Guide of the Hospital Survey On Patient Safety Culture (HSOPSC) questionnaire [37].

The distribution of participants and response rate (RR) of the intervention and control groups at M1, M2, and M3 are presented in the study flowchart (Figure 1).

**FIGURE 1:**
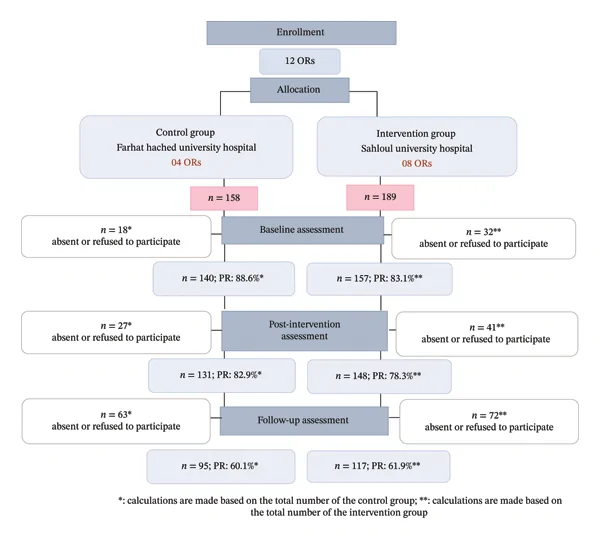
Study flowchart.

### 2.3. Study Instrument

Data for the baseline, postintervention, and follow‐up assessments were collected using the French version of the HSOPSC, developed by the Coordinating Committee for Clinical Evaluation and Quality in Aquitaine, France [38]. This French version was selected because it ensures better participant comprehension and has been widely used in previous studies conducted in the Tunisian healthcare setting [19, 20, 39, 40]. This version comprises 10 PSC dimensions and has demonstrated reliable psychometric properties. In the original validation study by Occelli et al. [38], the questionnaire’s overall Cronbach’s alpha was 0.88, and it ranged from 0.46 to 0.84 for the dimensions [38]. In the Tunisian context, the HSOPSC has been widely used in various hospital departments in Tunisia, including emergency, ORs, and intensive care units [19, 20, 39, 40]. The reliability of the tool was previously assessed in a sample of Tunisian OR caregivers, with Cronbach’s alpha for the entire questionnaire being 0.886 and ranging from 0.689 to 0.875 across its 10 dimensions [20]. In the current study, internal consistency was reassessed and showed satisfactory reliability, with an overall Cronbach’s alpha coefficient of 0.874 and dimension‐level coefficients ranging from 0.733 to 0.891.

Overall, the questionnaire consisted of 49 items: 40 items were used to measure ten dimensions related to PSC [37], two items assessed the overall perception of patient safety and the number of AEs reported during the last 12 months, and seven items captured the demographic and professional characteristics of participants. A 5‐point Likert scale was used to rate professionals’ agreement or disagreement (from 1 = don’t agree at all to 5 = strongly agree) and to assess frequency (from 1 = never to 5 = always) [37].

The HSOPSC includes items that are phrased both positively and negatively. For the purpose of analysis, only responses in favor of a developed PSC are considered positive. Responses with a score of 4 or 5 on the Likert scale are considered positive for items with positive wording. On the other hand, for items with negative wording, responses with a score of 1 or 2 are considered positive since they need to be reverse‐coded to calculate positive responses [37]. The HSOPSC does not provide an overall PSC score [19]. Thus, to calculate overall scores for the 10 dimensions, the average percentage of positive responses across the items of each dimension was calculated [37]. A dimension is deemed “to be improved” if its score falls at or below 50% [37]. Scores ranging between 51% and 74% are considered “poorly developed” [41]. Conversely, a dimension is classified as “developed” if its score equals or exceeds 75% [37].

### 2.4. Study Procedure

The study was conducted over a 21‐month period, comprising a 2‐month pre‐intervention phase, 15 months dedicated to the intervention, and four months for follow‐up. An overview of the study timeline and key phases is presented in Figure 2.

**FIGURE 2:**
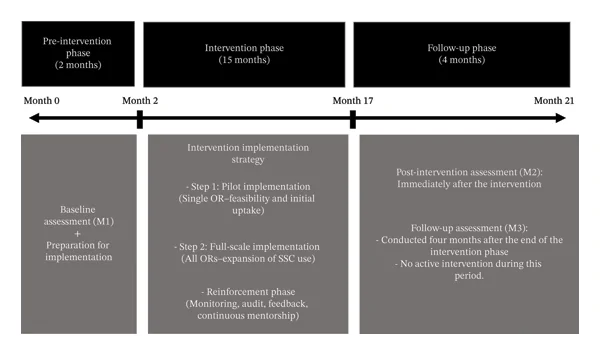
Overview of the study timeline.

#### 2.4.1. Pre‐Intervention Phase (Preparation Phase)

Prior to the SSC implementation, several preparatory activities were undertaken to ensure organizational readiness and facilitate the successful integration of the SSC into routine practice.

##### 2.4.1.1. Stakeholder Engagement

To secure the support and endorsement of administrative and clinical leaders, several meetings were held with the “OR Committee” and the “Quality Assurance Unit.” During these meetings, the research team provided a thorough explanation of the purpose of the SSC and emphasized its role in improving surgical patient safety and reducing AEs during surgical procedures.

##### 2.4.1.2. Contextual Adaptation of the SSC

In accordance with WHO recommendations, teams are encouraged to adapt the SSC to their organizational context [23]. Before implementation, two checklist items were modified to reflect the local context. First, the *Sign In* item was revised to include legal guardians in the verification process to accommodate pediatric patients and adults unable to provide consent. Second, during the COVID‐19 pandemic, a preoperative screening component was added in collaboration with infection prevention and control specialists.

Definition of the SSC rollout strategy: Key operational aspects of SSC implementation were defined prior to rollout, including the following: Who should be present in the OR? Who is responsible for administering the checklist? How should the checklist be conducted? *Can clinical activity continue during checklist administration? Where should the completed checklist be placed?*

##### 2.4.1.3. Preparation of Educational Materials

The principal investigator was responsible for preparing the intervention timeline in advance and all the necessary documents and training materials to support awareness. The educational package consisted of the SSC use manual, informational posters and flyers, lectures, brief educational sessions, unit‐based visits, etc.

##### 2.4.1.4. Baseline Assessments

Preliminary investigations included the baseline assessment (M1) of PSC in both groups (intervention and control). In addition, to gain a deeper understanding of the existing PSC, a qualitative study was performed to explore staff perceptions of surgical safety (13 individual interviews) [20].

#### 2.4.2. Intervention Phase

The intervention comprised three interrelated components as outlined in Table 1: the implementation of the WHO SSC as a mandatory structural change in perioperative practice, along with a set of educational and organizational strategies designed to support its adoption and sustained use.

**TABLE 1:** Detailed description of the multifaceted intervention components and their respective roles.

Component	Description	Specific activities	Expected role in influencing outcomes
1. Structural component	Mandatory integration of the SSC into routine perioperative workflow	‐ Contextual adaptation. ‐ Definition of rollout strategy (who leads, how, when, and where to store the SSC). ‐Mandatory documentation in the patient medical record.	‐Promotes standardization and accountability; ‐ Ensures consistent application across all surgical procedures.

2. Educational component	Training and skill‐building to ensure correct execution of the SSC	‐ 45‐min introductory training session. ‐ Supervised practice runs over 3 consecutive days. ‐ Biweekly 30–45‐min refresher sessions (3‐part format). ‐ Posters, flyers, door‐to‐door mini‐lectures. ‐ Certificates of completion.	‐ Builds knowledge, skills, and confidence; ‐ Promotes correct and consistent SSC execution.

3. Organizational component	Leadership, support systems, and reinforcement strategies	‐ Stakeholder engagement (OR Committee, Quality Assurance Unit). ‐ Nurse champions (peer coaching, on‐site support). ‐ One‐hour leadership training for OR managers. ‐ Ongoing mentorship (principal investigator, 3 visits/week). ‐ Audit and feedback at mid‐intervention (after 7 months) and postintervention. ‐ Addressing resistance (formal notice mandating SSC use).	‐ Creates an enabling environment; ‐ Sustains engagement; ‐ Addresses barriers; ‐ Reinforces accountability.

##### 2.4.2.1. Core Intervention: Implementation of the SSC

The core intervention consisted of implementing the WHO SSC as a standardized patient safety tool integrated into routine perioperative practice. The SSC is structured around three critical phases, *Sign In*, *Time Out*, and *Sign Out*, and requires active participation from all surgical team members [23].

The SSC was introduced as a mandatory component of surgical care across all participating ORs and was systematically integrated into routine perioperative workflow. Completion of the checklist was required for every surgical procedure and was documented in the patient’s medical record.

##### 2.4.2.2. Implementation Strategies (Educational and Organizational Components)

The implementation strategy was to start small and then expand following WHO recommendations [23]. Therefore, the intervention was carried out as follows:

**Step 1: Pilot phase (Neurosurgery OR)**

In line with WHO recommendations to initiate implementation by engaging motivated and willing teams [23], the intervention was first piloted in a volunteer OR (neurosurgery OR) to assess feasibility and refine implementation procedures. Following the pilot phase, the neurosurgery OR was integrated into the full‐scale implementation alongside the remaining seven ORs.

*Raising awareness*: The principal investigator conducted a structured introductory training session for all members of the neurosurgery surgical team. The session was approximately 45 min long and covered the rationale, benefits, and guidance on the practical use of the SSC, including a step‐by‐step application and documentation requirements. Supporting materials, including the SSC and the user manual, were provided to facilitate understanding and standardization of use.

*Practice runs:* Prior to full implementation, the neurosurgery OR team participated in supervised practice runs over three consecutive days, during which the research team was present during procedures to facilitate proper and consistent use of the SSC. These visits allowed the team to become familiar with integrating the SSC into daily practice, clarify uncertainties, and build confidence in its application. Once the neurosurgical team demonstrated sufficient confidence and proficiency with the process, the intervention was scaled up to the remaining seven ORs for full implementation.

**Step 2: Full-scale implementation in other ORs.**

*Dissemination of positive preliminary feedback on SSC implementation*: Positive experiences and preliminary feedback from the pilot phase were disseminated across all ORs to support acceptance and uptake of the SSC.

*Educational content and delivery methods*: The goal of the educational program was to ensure that everyone involved in surgical procedures understood that the SSC was the new expected “standard of care”. Multiple approaches were used to educate perioperative staff. The implementation package used in the pilot phase was maintained and expanded to ensure consistency across all sites.

A standardized 45‐min introductory training session, identical in content to that delivered in the pilot phase, was conducted by the principal investigator for all perioperative staff. This standardized approach ensured that all OR staff had a common understanding of the SSC objectives, structure, and execution. In addition, similar to the pilot phase, supervised practice runs were also conducted in the remaining ORs to facilitate real‐time integration and guidance.

Multiple strategies were employed to reinforce learning and support continued use, including continuous visual reminders and reference materials. Each OR displayed two large posters summarizing SSC objectives and its role in preventing surgical AEs. Flyers were distributed outlining key operational elements, including team composition during checklist execution, responsibility allocation, procedural steps, and documentation requirements.

To ensure an interprofessional approach to SSC implementation, the target population included all perioperative professionals, i.e., surgeons, anesthesiologists, nurses, OR specialized nurses, and assistant caregivers.

*Leadership engagement and local nurse champions*: To strengthen OR managers’ commitment to the project, a one‐hour training session was conducted for managers during which recent research findings were presented on the high prevalence of AEs associated with surgical care. The training also highlighted the essential role of managers in the successful implementation of the project and the importance of their leadership and genuine commitment to sustainability.

In addition, designated nurse champions were appointed in each OR. Their responsibilities included peer coaching, on‐site support during SSC execution, reinforcement of correct practice, and serving as local points of contact for implementation‐related issues. Importantly, they received training on the SSC procedures and their coaching responsibilities before assuming their roles.

##### 2.4.2.3. Ongoing Support, Monitoring, and Reinforcement

After introducing the SSC to all ORs, active engagement of OR staff was maintained through structured monitoring and reinforcement activities.

*Ongoing mentorship:* The principal investigator provided continuous support through regular visits (three per week) in collaboration with local nurse champions. This approach facilitated ongoing mentorship, feedback, and reinforcement of SSC integration into routine practice.

*Addressing resistance*: During the implementation phase, some OR professionals perceived the change as unnecessary and resisted using the SSC. To address this, the research team administered additional training and support. In addition, a formal notice was prominently displayed in all ORs, emphasizing that SSC use was legally mandatory and that it must be completed and included in each patient’s medical record.

*Ongoing education:* Regular training, complemented by visual reminders, was provided. For instance, new posters were displayed in all ORs to remind staff of the 12 key points required for proper execution of the SSC.

During the second half of the intervention, bi‐weekly refresher training sessions were carried out in a 3‐part format, each lasting 30–45 min. These sessions emphasized the correct SSC execution and stressed the importance of verbalization. The three refresher sessions each had a distinct focus: (1) a review of SSC steps; (2) a demonstration of successful verbalization approaches; and (3) a question‐and‐answer session to address challenges and clarify any ambiguities. The training sessions were facilitated by the principal investigator and the local nurse champions. All perioperative staff, including surgeons, anesthesiologists, nurses, OR specialized nurses, and assistant caregivers, were required to attend. Attendance was tracked, and certificates of completion were awarded to those who attended the training sessions.

*Audit and feedback*: SSC adherence was monitored throughout the intervention period. SSC compliance and completion were evaluated at different time points to ensure that the implementation effectively met its goals. A 7‐month SSC compliance report was prepared halfway through the intervention to assess progress in implementation. At the end of the intervention, a summary report was drafted and distributed to all ORs, providing feedback on SSC adherence, trends in compliance rates over the course of the 15 months, and identification of the OR with the highest compliance rate.

Overall, the intervention followed a standardized, stepwise protocol based on WHO recommendations, ensuring high intervention fidelity and implementation consistency across all participating ORs throughout the study period. This structured approach facilitates replicability in similar perioperative settings. Regular monitoring visits, direct observations of SSC use, continuous mentorship, and periodic audits of checklist completion were conducted to maintain fidelity. Feedback from these activities was used to identify barriers and reinforce adherence to protocol.

Despite these efforts to maintain fidelity, the intervention took place during the COVID‐19 pandemic, which presented organizational and logistical challenges, such as increased workload, variability in surgical activity, and limited access to ORs. Adaptations included flexible scheduling of educational sessions, training in smaller groups, and greater involvement of nurse champions for on‐site peer coaching and mentoring. Importantly, monitoring and reinforcement activities were maintained in accordance with infection prevention measures.

#### 2.4.3. Post‐Intervention Phase

Fifteen months after the intervention began, the HSOPSC was re‐administered to all staff in the participating ORs, in both the intervention and control groups. Subsequently, a 4‐month follow‐up period took place, during which the sustainability of the intervention’s outcomes was assessed without performing any additional visits, awareness activities, education, or training. At the end of this period, the HSOPSC was once again administered to OR professionals in both groups.

### 2.5. Data Analysis

Prior to data entry, all questionnaires were checked for completeness. All incomplete questionnaires were removed from the dataset and excluded from the analysis. Then, data were coded, entered, and analyzed using the Statistical Package for Social Sciences, version 26 (IBM, SPSS Inc., Chicago, IL, USA). Descriptive statistics were used to summarize sample characteristics, including frequencies (*n*) and percentages (%) for qualitative variables. Means (*M*) and standard deviations (SD) were used for quantitative data. The internal reliability of the HSOPSC was assessed with Cronbach’s *α* coefficient.

Data were analyzed using a population‐averaged approach appropriate for quasiexperimental controlled before–after designs with repeated measurements across time. Comparisons between intervention and control groups over time were performed using multivariate techniques to assess overall trends in PSC dimensions.

The intervention was implemented at the hospital level, and outcomes were assessed at multiple time points using independent cross‐sectional samples of healthcare professionals. While the hierarchical structure of the data (professionals nested within ORs and hospitals) was acknowledged, the analytical approach focused on estimating overall between‐group and over‐time effects at the population level.

To calculate overall scores for the 10 dimensions, the average percentage of positive responses across the items of each dimension was calculated. To determine the changes in mean scores of each PSC dimension in the two groups over the three time points (M1, M2, and M3), the two‐way multivariate analysis of variance (two‐way MANOVA) was used.

The normality of data was assessed using the Kolmogorov–Smirnov test, and Levene’s test of equality of variances was used to test the assumption of homogeneity of variances. Additionally, the Bonferroni post hoc test for adjustment of multiple comparisons was used for the fixed factor “time” (M1, M2, and M3) to assess score variations across the three measurement time points. A chi‐square test was employed to examine differences between response categories for the two items “patient safety level” and “number of AEs reported.” The level of significance was set at 5%.

### 2.6. Ethical Considerations

Before data collection, the institutional ethics committee of the Faculty of Medicine of Sousse reviewed and approved the study protocol (reference number: CEFMS 31/2019). In addition, to get access to the study sites, administrative authorizations were obtained from the OR department heads. The study objectives and procedures were explained to participants. They were informed that participation was completely voluntary and that they could refuse or withdraw from the study at any time without any consequences.

Participant confidentiality and data anonymity were strictly maintained throughout the study. All collected data were coded and stored securely, and no personal identifiers were used during analysis or reporting.

## 3. Results

The study comprised three stages of data collection. In the baseline assessment (M1), a total of 297 out of 347 eligible OR professionals participated in the study, resulting in an RR of 85.6%.

### 3.1. Participant Characteristics

In both control and intervention groups, the majority of the participants were female and belonged to the nursing staff. Among the OR professionals who participated in the baseline, postintervention, and follow‐up assessments, the mean age ranged from 39.7 (±7.28) to 40.4 (±7.59) years. Detailed participant characteristics are presented in Table 2.

**TABLE 2:** Participants’ characteristics of the intervention and control groups.

	Control group			Intervention group		
	Baseline assessment M1	Postintervention assessment M2	Follow‐up assessment M3	Baseline assessment M1	Postintervention assessment M2	Follow‐up assessment M3
Characteristics						
Age (mean ± SD)	39.7 (±7.28)	40.3 (±7.67)	40.4 (±7.59)	39.7 (±7.67)	40.1 (±7.19)	40.2 (±7.61)
	*n* (%)	*n* (%)	*n* (%)	*n* (%)	*n* (%)	*n* (%)

*Gender*						
Male	54 (38.6)	52 (39.7)	39 (41.1)	70 (44.6)	58 (39.2)	44 (37.6)
Female	86 (61.4)	79 (60.3)	56 (58.9)	87 (29.3)	90 (60.8)	73 (62.4)

*Professional title*						
Physicians	44 (31.4)	34 (26)	25 (26.3)	47 (29.9)	39 (26.4)	26 (22.2)
Nurses	31 (22.2)	33 (25.2)	29 (30.5)	60 (38.2)	58 (39.2)	50 (42.7)
OR nurses	64 (45.7)	61 (46.6)	39 (41.1)	47 (29.9)	48 (32.4)	37 (31.6)
Assistant caregivers	1 (0.7)	3 (2.2)	2 (2.1)	3 (2)	3 (2)	4 (3.5)

*Work experience*						
< 10 years	80 (57.1)	75 (57.3)	50 (52.6)	79 (50.3)	81 (54.7)	63 (53.8)
> 10 years	60 (42.9)	56 (42.7)	45 (47.4)	78 (49.7)	67 (45.3)	54 (46.2)

*Working years in OR*						
< 5 years	62 (42.3)	58 (44.3)	41 (43.2)	56 (35.7)	49 (33.1)	34 (29.1)
> 5 years	78 (55.7)	73 (55.7)	54 (56.8)	101 (64.3)	99 (66.9)	83 (70.9)

*Involvement in risk management committees*						
Yes	26 (18.6)	26 (19.8)	18 (18.9)	40 (25.5)	37 (25)	29 (24.8)
No	114 (81.4)	105 (80.2)	77 (81.1)	117 (74.5)	111 (75)	88 (75.2)
Total	140 (100)	131 (100)	95 (100)	157 (100)	148 (100)	117 (100)

### 3.2. Associations Between the Intervention and the Overall Patient Safety Level and the Number of Reported AEs in the Previous 12 Months

The intervention implemented at Sahloul University Hospital was associated with a significant improvement in the overall patient safety level in the intervention group (Table 3). Prior to the intervention, 70.7% (*n* = 111) of participants rated the overall patient safety level as “Excellent,” “Very good,” and “Acceptable.” Following the intervention, positive perceptions of patient safety increased to 82.4% (*n* = 122), and this improvement was sustained at follow‐up with 81.2% (*n* = 95) of respondents reporting positive perceptions. The change was statistically significant, *p*(M1–M2) = 0.016; *p*(M1–M3) = 0.04. No significant changes were observed in the control group.

**TABLE 3:** Associations between the intervention and the overall patient safety level and the number of reported AEs in the past 12 months in the intervention and control groups.

Group	Overall patient safety level	M1	M2	M3	*p* value (M1‐M2)	*p* value (M1‐M3)
		*n* %	*n* %	*n* %		
Intervention group	Excellent/very good/acceptable	111 (70.7)	122 (82.4)	95 (81.2)	0.016	0.04
	Poor/failing	46 (29.3)	26 (17.6)	22 (18.8)	0.01	0.04
	Total	157 (100)	148 (100)	117 (100)		

Control group	Excellent/very good/acceptable	85 (60.7)	71 (54.2)	47 (49.5)	0.28	0.09
	Poor/failing	55 (39.3)	60 (45.8)	48 (50.5)	0.2	0.09
	Total	140 (100)	131 (100)	95 (100)		

**Group**	**AEs reporting (in the past 12 months)**					

Intervention group	None	126 (80.3)	106 (71.6)	91 (77.8)	0.075	0.6
	At least once	31 (19.7)	42 (28.4)	26 (22.2)	0.075	0.6
	Total	157 (100)	148 (100)	117 (100)		

Control group	None	129 (92.1)	113 (86.3)	81 (85.3)	0.12	0.09
	At least once	11 (7.9)	18 (13.7)	14 (14.7)	0.12	0.09
	Total	140 (100)	131 (100)	95 (100)		

Regarding the number of AEs reported during the previous 12 months, no significant changes were observed in either the intervention or the control group.

### 3.3. Baseline Comparison of PSC Dimensions Between the Intervention and Control Groups

At baseline (M1), no statistically significant differences were observed between the intervention and control groups across the 10 PSC dimensions (all *p* values ranged between 0.06 and 0.89). Both groups showed comparable scores across all PSC dimensions. “Teamwork within units” (D5) showed the highest scores in both the intervention (47.6%) and control (42.9%) groups, whereas “Non‐punitive response to errors” (D7) had the lowest scores in both groups (23.8% and 21.9%, respectively).

### 3.4. Summary of the Intervention’s Associated Improvements on the 10 PSC Dimensions

Overall, the intervention was associated with improvements in four PSC dimensions, namely “Organizational learning‐continuous improvement” (D4), “Teamwork within units” (D5), “Communication openness” (D6), and “Teamwork across units” (D10). The observed improvements were sustained at the follow‐up assessment, as shown in Figure 3.

**FIGURE 3:**
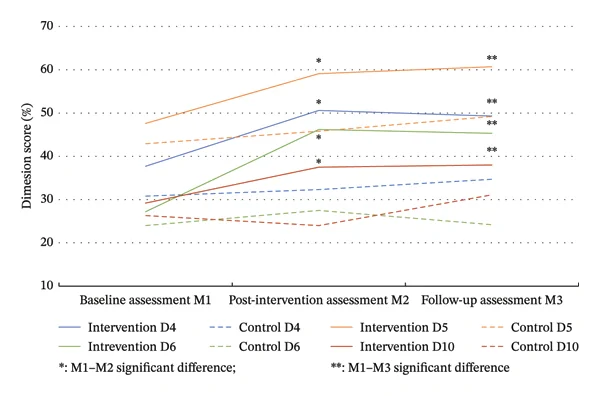
Trends in improved PSC dimension scores across M1, M2, and M3.

Table 4 summarizes the associations between the intervention and the 10 PSC dimensions in the intervention and control groups across three time points (M1, M2, and M3).

**TABLE 4:** A summary of the associations between the intervention and the 10 PSC dimensions.

PSC dimensions	Group	Baseline assessment M1 (%)	Postintervention assessment M2 (%)	Follow‐up assessment M3 (%)	Multiple comparisons	*p* value^α^
D1	Intervention	38.1	44.9	41.5	M1–M2	0.111
					M1–M3	1.000
					M2–M3	0.962
	Control	28.8	32.3	32.9	M1–M2	0.927
					M1–M3	0.813
					M2–M3	1.000

D2	Intervention	25.9	34.9	34.2	M1–M2	0.077
					M1–M3	0.162
					M2–M3	1.000
	Control	25.2	28.8	26	M1–M2	1.000
					M1–M3	1.000
					M2–M3	1.000

D3	Intervention	41.4	43.9	39.7	M1–M2	1.000
					M1–M3	1.000
					M2–M3	0.851
	Control	30.9	32.3	35.5	M1–M2	1.000
					M1–M3	0.805
					M2–M3	1.000

D4	Intervention	**37.7**	**50.6**	**49.3**	**M1–M2**	**< 10^−3^**
					**M1–M3**	**0.001**
					M2–M3	1.000
	Control	30.8	32.3	34.7	M1–M2	1.000
					M1–M3	0.788
					M2–M3	1.000

D5	Intervention	**47.6**	**59.1**	**60.7**	**M1–M2**	**0.004**
					**M1–M3**	**0.002**
					M2–M3	1.000
	Control	42.9	45.8	49.2	M1–M2	1.000
					M1–M3	0.386
					M2–M3	1.000

D6	Intervention	**27.2**	**46.2**	**45.3**	**M1–M2**	**< 10^−3^**
					**M1–M3**	**< 10^−3^**
					M2–M3	1.000
	Control	24	27.5	24.2	M1–M2	1.000
					M1–M3	1.000
					M2–M3	1.000

D7	Intervention	23.8	30.4	28.5	M1–M2	0.138
					M1–M3	0.548
					M2–M3	1.000
	Control	21.9	24.2	20	M1–M2	1.000
					M1–M3	1.000
					M2–M3	0.854

D8	Intervention	*25.9*	*12.6*	*9.7*	*M1–M2*	*< 10^−3^*
					*M1–M3*	*< 10^−3^*
					M2–M3	0.931
	Control	*28.6*	*17.6*	*12.6*	*M1–M2*	*< 10^−3^*
					*M1–M3*	*< 10^−3^*
					M2–M3	0.352

D9	Intervention	32	34.8	30.9	M1–M2	1.000
					M1–M3	1.000
					M2–M3	0.737
	Control	*30.9*	*23.1*	*18.4*	M1–M2	0.053
					*M1–M3*	*0.002*
					M2–M3	0.6

D10	Intervention	**29.2**	**37.5**	**38**	**M1–M2**	**0.015**
					**M1–M3**	**0.015**
					M2–M3	1.000
	Control	26.3	24	31.1	M1–M2	1.000
					M1–M3	0.483
					M2–M3	0.125

*Note:* Values in bold refer to a significant positive change; values in italic refer to a significant negative change.

^α^Adjusted with Bonferroni for multiple comparisons.

After SSC implementation, the “teamwork within units” (D5) showed the greatest improvement at both M2 and M3. The baseline score was 47.6% and, following the intervention, the score significantly increased to 59.1% (*p* = 0.004). This improvement was sustained at follow‐up (60.7%, *p* = 0.002).

## 4. Discussion

Recent studies have highlighted the need to improve safety culture in surgical units, yet interventions to enhance it in these settings remain limited [7, 27]. In the present study, the multifaceted SSC intervention was associated with significant improvements in PSC dimensions. In contrast, no significant changes were observed in the control group, likely reflecting the absence of a structured intervention and limited targeted support.

To better understand these findings, it is important to consider the key determinants of PSC. PSC is a multidimensional construct shaped by educational, organizational, and systemic factors [42]. In line with this perspective, the intervention in this study integrated three complementary components: structural (implementation of the SSC as a mandated tool), educational (training and awareness campaigns), and organizational (leadership engagement, nurse champions, mentorship, and audit and feedback). Therefore, the observed improvements are unlikely to be attributable to any single component; rather, they likely reflect the combined and synergistic effects of these elements.

Our findings suggest that the multifaceted intervention may have contributed to improvements in the overall perceived patient safety level within the intervention group, as reflected by the positive shift in participants’ perceptions. These findings are consistent with previous studies [43–45]. For instance, a randomized controlled trial provided additional evidence of improved patient safety perceptions following the implementation of the WHO SSC [44]. According to Verbeek‐van Noord et al., the perceptions of caregivers regarding patient safety in their units may provide valuable insights [46]. In a review of ethnographic studies, Dixon‐Woods highlighted that ORs where caregivers and managers demonstrated higher scores on safety climate surveys were associated with lower rates of adverse patient safety indicators [47].

Undoubtedly, the OR is a dynamic and high‐pressure work setting in which a diverse team of healthcare professionals performs complex tasks that demand both technical expertise and nontechnical skills [43]. Within this environment, they must simultaneously process real‐time information from the patient, colleagues, and monitoring devices. However, the fast‐paced and complex nature of the OR can also pose inherent challenges that increase the likelihood of errors [1, 2, 43].

Within this context, the implementation of the SSC serves as a valuable tool to promote consistency in surgical team performance during critical moments [48]. In addition to its practical benefits, the SSC facilitates effective communication and fosters collaborative teamwork [48]. It plays a vital role in shaping the overall safety climate within the surgical environment [49]. Therefore, SSC implementation sends a clear message to all team members that patient safety is paramount and should be prioritized at all times [48, 49].

Despite the improvement in patient safety perceptions in the intervention group, the number of reported AEs did not improve. This divergence suggests that while the intervention effectively strengthened safety culture perceptions, it did not sufficiently address structural and behavioral barriers to incident reporting. In particular, underreporting in surgical settings is often influenced by fear of blame, limited feedback following incident submission, concerns about professional consequences, and time constraints [50]. These factors may not be easily modified through the SSC implementation alone. Therefore, although participants had more positive safety culture perceptions, it did not necessarily translate into changes in reporting behavior. However, one study reported a decrease in the number of reported events following SSC implementation [51], which the authors suggested may reflect a reduction in near‐misses due to improved team preparation and workflow standardization, rather than changes in reporting behavior [51]. Future interventions should incorporate targeted strategies specifically aimed at improving reporting practices, such as strengthening nonpunitive reporting systems, ensuring timely feedback, and reinforcing leadership support to promote learning from errors and shifting away from punitive cultures [52].

Our intervention was associated with notable improvements in four PSC dimensions: “organizational learning‐continuous improvement” (D4), “teamwork within units” (D5), “communication openness” (D6), and “teamwork across units” (D10). These improvements were also sustained at follow‐up.

Notably, most interventional studies aimed at improving PSC in ORs were conducted in high‐income countries (HICs). HICs generally have more resources and funding available for research [53, 54]. Additionally, HICs often have more established healthcare systems with greater infrastructure, a highly trained workforce, standardized procedures for ensuring patient safety, and higher levels of awareness and prioritization of PSC among healthcare professionals and policymakers [53].

Conversely, there remains a notable scarcity of studies specifically addressing the improvement of PSC in ORs within LMICs [32, 33]. Multiple interrelated factors contribute to this gap, including resource constraints such as limited staffing and inadequate technological infrastructure, as well as resistance to change and insufficient awareness among healthcare providers [53, 55, 56]. These challenges collectively limit the capacity of hospitals in LMICs to implement robust patient safety protocols [53, 55]. In Tunisia, these structural and organizational constraints are particularly pronounced. Factors such as limited staffing, variable access to training resources, and less mature patient safety reporting systems may influence both baseline levels of PSC and the extent of improvement achievable through interventions. Furthermore, variations in organizational readiness, leadership engagement, and access to continuous professional development opportunities can shape implementation processes and outcomes.

As such, successful implementation in low‐resource settings often requires context‐sensitive adaptation, flexibility in delivery, and the use of locally embedded strategies, such as nurse champions and ongoing mentorship, rather than direct replication of models developed in HICs.

Haugen et al. [22] conducted a similar study in Norway and reported that eight of the 12 PSC dimensions improved significantly after the intervention [22]. The difference between the outcomes of our study and those of the Norwegian study may be explained by the fact that the SSC had already been implemented since 2010 in the Norwegian context [22], whereas in our context, the surgical staff in the intervention hospital had no prior experience using it.

The COVID‐19 pandemic introduced further contextual challenges. Postponing the intervention was discussed, given the major shift in healthcare priorities and resource limitations [57]. However, the research team decided to proceed, with necessary adaptations, in line with evidence that quality improvement should continue during crises [58, 59]. The implementation of this intervention occurred under challenging circumstances, creating several contextual constraints that may have influenced both its execution and the study outcomes. At the organizational level, the pandemic led to increased workloads, redeployment of staff, and variations in surgical activity, mainly due to the postponement of elective procedures [57]. In addition, infection prevention measures such as limited access to clinical areas and restrictions on gatherings impacted the feasibility of training activities, supervision, and routine monitoring activities.

Despite these challenges, adaptive strategies were implemented to ensure the intervention’s continuity. Training sessions were rearranged into smaller groups and were delivered flexibly. Nurse champions played a pivotal role in providing on‐site support, increasing adherence, and allowing peer coaching. Monitoring and feedback processes were maintained but adapted to the changing context. These mitigation strategies were likely important in maintaining engagement with the intervention in the face of the pressure posed by the pandemic.

Encouragingly, the intervention was associated with significant and sustained improvements in PSC, suggesting that SSC implementation can succeed even during crisis. The use of a concurrent control group also strengthens the interpretation of these results, as it allows differentiation between the intervention’s associated improvements and the broader system disruption associated with the pandemic.

Beyond the improvements observed in PSC dimensions, our study highlighted the value of empowering nurses as leaders and champions of patient safety initiatives. Our nurse‐led approach may have supported nurses in becoming proactive facilitators of the SSC, rather than passive participants. This aligns with the WHO’s call to focus on nursing leadership in order to build resilient health systems, particularly in resource‐limited settings [60]. For instance, the role of the “nurse champion” was previously recognized as an important success factor in implementing complex interventions [61]. Nurses provide essential peer influence and sustained advocacy, helping to integrate new practices into clinical routines and overcome resistance [61]. Our findings suggest that nurses can act as effective change agents when given the appropriate support and leadership opportunities.

### 4.1. Strengths and Limitations

This study has several strengths. First, the quasiexperimental design included a concurrent control group and spanned 21 months, strengthening confidence in the observed associations. Second, the intervention was associated with significant improvements in PSC that were sustained at follow‐up. Third, the intervention provided nurses with a structured platform to exercise leadership, as they played a crucial role in championing the SSC and facilitating team communication. Fourth, RR remained high across all three assessments (ranging from 78.3% to 88.6%), exceeding the minimum threshold for representativeness and supporting an accurate reflection of OR professionals’ perceptions. Finally, the successful execution of this initiative during the COVID‐19 pandemic underscores the feasibility and resilience of nurse‐led quality improvement even under challenging circumstances.

Nevertheless, there are several limitations that must be acknowledged. First, the lack of randomization may have led to uncontrolled confounding, limiting causal inference. Moreover, despite the absence of significant differences in PSC measures between the intervention and control groups at baseline, potential differences between the two hospitals, such as staffing patterns, structural characteristics, and organizational culture, were not fully controlled for and may have influenced the observed outcomes.

In addition, selection bias cannot be completely excluded despite the use of an exhaustive recruitment strategy. Furthermore, like any survey‐based study, self‐reporting bias may have influenced responses, despite assurances of anonymity.

While all HSOPSC dimensions demonstrated acceptable internal consistency, the average percentage of positive responses should be interpreted as reflecting the staff’s PSC perceptions rather than objective measures of organizational performance.

Additionally, multi‐level or cluster‐adjusted analyses were not performed to explicitly account for possible clustering of responses within ORs and hospitals, as it was not feasible to collect the required data for such analyses at the time of data collection, which may have affected variance estimates.

Finally, the quasiexperimental design of the study makes it susceptible to secular trends and external system‐level changes over time. In particular, the COVID‐19 pandemic may have introduced additional contextual influences regarding workload, staffing, and organizational priorities, which could not be fully controlled, despite the inclusion of a concurrent control group.

### 4.2. Implications for Nursing Management

The findings of this study provide important implications for nurses and perioperative nursing leaders interested in improving PSC in resource‐limited settings. The successful implementation of the SSC may require more than introducing a standardized tool; it depends on strong leadership commitment, organizational support, and the empowerment of frontline healthcare professionals. Nurse managers should consider developing and supporting local nurse champions who can facilitate the integration of the SSC, promote team engagement, provide peer support, and serve as role models for safety practices.

OR managers and nursing leaders have an essential role in creating a favorable environment to support a sustainable implementation. This includes ensuring dedicated time for training activities, providing continuous mentorship, encouraging interprofessional participation, and establishing regular audit and feedback processes to monitor adherence and identify areas for improvement. This structured approach can help transform the SSC from a procedural requirement into an embedded component of daily surgical practice.

In resource‐limited settings, nurse managers should also anticipate barriers related to workload pressure, resistance to change, and limited safety infrastructure. Overcoming these challenges requires transparent communication, active staff involvement in improvement initiatives, and the promotion of a nonpunitive reporting culture where healthcare professionals can feel safe to report incidents and near‐misses. Strengthening feedback mechanisms and encouraging learning from errors rather than blame are essential strategies to foster continuous improvement and sustain a positive PSC.

## 5. Conclusions

This study suggests that a multifaceted intervention, combining the implementation of the WHO SSC with team‐based training, ongoing mentorship, and nurse‐led facilitation, was associated with improvements in four PSC dimensions, including “Organizational learning‐continuous improvement” (D4), “Teamwork within units” (D5), “Communication openness” (D6), and “Teamwork across units” (D10). No significant changes were observed in AE reporting. These improvements reflect OR professionals’ perceptions of PSC rather than objective patient safety outcomes.

The findings suggest that context‐specific approaches may contribute to the integration of structured safety practices into daily surgical practice and highlight the importance of nursing leadership in advancing patient safety. Empowering nurses as local SSC champions in the OR may facilitate compliance, collaboration, and sustainable change, especially in LMICs.

Notably, the intervention’s successful implementation during the COVID‐19 pandemic suggests that nurse‐led quality improvement initiatives can remain feasible even under challenging conditions.

Ultimately, sustainable progress in patient safety requires coordinated leadership, interprofessional collaboration, and the strategic empowerment of nurses as key drivers of quality and safety.

**Nomenclature**
AEs: Adverse events
HICs: High‐income countries
HSOPSC: Hospital survey on patient safety culture
IOM: Institute of Medicine
KTA: Knowledge to action
LMICs: Low‐ and middle‐income countries
OR: Operating room
PSC: Patient safety culture
RR: Response rate
SSC: Surgical safety checklist
SSI: Surgical site infections
SPO: Structure–process–outcome
WHO: World Health Organization

## Author Contributions

Conceptualization: Wiem Aouicha, Mohamed Ayoub Tlili, Manel Mallouli, and Houyem Said Latiri. Methodology: Wiem Aouicha, Mohamed Ayoub Tlili, Houyem Said Latiri, Manel Mallouli, and Nick Sevdalis. Investigation: Wiem Aouicha and Mohamed Ayoub Tlili. Project administration: Wiem Aouicha, Houyem Said Latiri, and Manel Mallouli. Data curation and formal analysis: Wiem Aouicha, Mohamed Ayoub Tlili, and Manel Mallouli. Writing–original draft: Wiem Aouicha. Writing–review and editing: Wiem Aouicha, Mohamed Ayoub Tlili, Bushra Alshammari, Hamdi Lamine, Nahed Mousa Saber, Nojoud Abdullah Alrashidi, Areej Ali Fahad Alshammary, Hadya Obied Alenzi, Samah Ramadan Ibrahim Elrefaey, Mohammad Alboliteeh, Areej Ali Fahad Alshammary, Hind mobark fahd Alshamry, Maha Sanat Alrashedi, Houyem Said Latiri, Manel Mallouli, Nick Sevdalis, and Aziza Zakaria Faramawy Ali. Supervision: Wiem Aouicha, Manel Mallouli, Nick Sevdalis, Houyem Said Latiri, and Aziza Zakaria Faramawy Ali.

## Funding

Funding from the Scientific Research Deanship at University of Ha’il, Saudi Arabia, has been received to conduct this research.

## Conflicts of Interest

Nick Sevdalis is the director of London Safety and Training Solutions Ltd., which offers training and improvement and implementation solutions to healthcare organizations and the pharmaceutical industry. The other authors declare no conflicts of interest.

## Data Availability

The data supporting the findings of this study, including the adapted SSC implementation materials and training resources, are available from the corresponding author upon reasonable request. Access is subject to approval by the hospital’s Quality Assurance Unit, in accordance with institutional policies.
